# MHC class II variation in a rare and ecological specialist mouse lemur reveals lower allelic richness and contrasting selection patterns compared to a generalist and widespread sympatric congener

**DOI:** 10.1007/s00251-015-0827-4

**Published:** 2015-02-18

**Authors:** Eva Pechouskova, Melanie Dammhahn, Markus Brameier, Claudia Fichtel, Peter M. Kappeler, Elise Huchard

**Affiliations:** 1 Behavioral Ecology and Sociobiology Unit, German Primate Center, Kellnerweg 4, Göttingen, Germany; 2 Animal Ecology, University of Potsdam, Maulbeerallee 1, 14469 Potsdam, Germany; 3 Primate Genetics Laboratory, German Primate Center, Kellnerweg 4, Göttingen, Germany; 4 Department of Sociobiology/Anthropology, Johann-Friedrich-Blumenbach Institut für Zoologie & Anthropologie Universität Göttingen, Kellnerweg 6, 37077 Göttingen, Germany; 5 CEFE-CNRS, 1919 Route de Mende, 34295 Montpellier Cedex 5, France

**Keywords:** Primates, Cheirogaleidae, *Microcebus berthae*, 454 pyrosequencing

## Abstract

The polymorphism of immunogenes of the major histocompatibility complex (MHC) is thought to influence the functional plasticity of immune responses and, consequently, the fitness of populations facing heterogeneous pathogenic pressures. Here, we evaluated MHC variation (allelic richness and divergence) and patterns of selection acting on the two highly polymorphic MHC class II loci (DRB and DQB) in the endangered primate Madame Berthe’s mouse lemur (*Microcebus berthae*). Using 454 pyrosequencing, we examined MHC variation in a total of 100 individuals sampled over 9 years in Kirindy Forest, Western Madagascar, and compared our findings with data obtained previously for its sympatric congener, the grey mouse lemur (*Microcebus murinus*). These species exhibit a contrasting ecology and demography that were expected to affect MHC variation and molecular signatures of selection. We found a lower allelic richness concordant with its low population density, but a similar level of allelic divergence and signals of historical selection in the rare feeding specialist *M. berthae* compared to the widespread generalist *M. murinus*. These findings suggest that demographic factors may exert a stronger influence than pathogen-driven selection on current levels of allelic richness in *M. berthae*. Despite a high sequence similarity between the two congeners, contrasting selection patterns detected at DQB suggest its potential functional divergence. This study represents a first step toward unravelling factors influencing the adaptive divergence of MHC genes between closely related but ecologically differentiated sympatric lemurs and opens new questions regarding potential functional discrepancy that would explain contrasting selection patterns detected at DQB.

**Electronic supplementary material:**

The online version of this article (doi:10.1007/s00251-015-0827-4) contains supplementary material, which is available to authorized users.

## Introduction

The island of Madagascar, one of the world’s biodiversity hotspots, has faced rapid deforestation over the last century, resulting in population fragmentation of many endemic primates (Lemuriformes) (Ganzhorn et al. 2001; Mittermeier et al. 1992; Schwitzer et al. 2013a, b), 94 % of which are currently classified as threatened (Schwitzer et al. 2013a). Many of these species are highly arboreal, inhabit restricted biogeographic ranges and exhibit fast life histories and higher population turnover compared to most anthropoid primates; however, the average life span is thought to be compromised by high extrinsic mortality pressure in the wild populations (Kraus et al. 2008; Fichtel 2012; Kappeler 2012). These characteristics make lemurs particularly vulnerable to an ongoing habitat degradation that may disrupt gene flow and cause local demographic fluctuations. This may in turn result in an irreversible loss of genetic diversity in lemurs, especially those species with restricted spatial distribution. Given the ongoing rate of deforestation, rapid surveys of remaining genetic diversity and its potential consequences for the future viability of these populations are essential to determine conservation priorities (Kremen et al. 2008). Although difficult to detect in small populations (Chikhi et al. 2010), signals of demographic or genetic bottlenecks or decreasing genetic diversity at neutral markers seem present across lemur taxa (Louis et al. 2005; Olivieri et al. 2008; Markolf et al. 2008; Radespiel et al. 2008; Craul et al. 2009; Razakamaharavo et al. 2010; Holmes et al. 2013; Baden et al. 2014). However, little is known regarding its potential fitness consequences and the capacity of such populations to maintain an effective level of functional (adaptive) genetic variation (allelic richness and divergence), sufficient to ensure their health and survival.

Genes of the major histocompatibility complex (MHC) are well suited to study the adaptive maintenance of genetic variation given their immune function (Klein 1986) and extreme polymorphism (e.g. Hedrick 2002; Garrigan and Hedrick 2003; Sommer 2005; Piertney and Oliver 2006; Spurgin and Richardson 2010). MHC genes are coding for MHC molecules (cell surface glycoproteins) that trigger the immune response by presenting antigens at antigen-binding sites (ABS) to T-lymphocytes, which then activate further components of the immune system. Each MHC molecule can bind only a limited array of antigens, and a number of mechanisms may increase the spectrum of antigens recognised, including gene duplications, extensive allelic diversity, polymorphism at ABS (Hughes and Nei 1988), or polymorphism outside ABS that may alter the 3D positioning of ABS contact residues (Bjorkman and Burmeister 1994).

Balancing selection, primarily exerted by pathogen pressure, is thought to represent one of the major forces driving MHC polymorphism (reviewed in Bernatchez and Landry 2003; Sommer 2005; Piertney and Oliver 2006; Milinski 2006; Spurgin and Richardson 2010). Three non-mutually exclusive evolutionary mechanisms have been proposed to explain this polymorphism. First, due to the codominant expression of MHC alleles and the function of MHC molecules in the immune response, MHC heterozygous individuals may be at an advantage in a population facing heterogeneous pathogen pressures (i.e. heterozygote advantage; Doherty and Zinkernagel 1975). Second, MHC alleles that are advantageous in protection against dominant pathogens in a given environment (Apanius et al. 1997) may temporarily rise in frequency, until pathogens evolve resistance to the most common host alleles, which are then progressively replaced by rarer alleles (i.e. frequency-dependent selection; Snell 1968; Bodmer 1972; Borghans et al. 2004). Finally, parasite communities typically vary in space and time and may thereby select distinct sets of MHC alleles in host populations (i.e. fluctuating selection; Hedrick 1999; Spurgin and Richardson 2010; Eizaguirre et al. 2009, 2012). As a result, patterns of selection on MHC genes are likely to be durably affected by the diversity and frequency of MHC alleles present in the host population and, hence, by its demography (Hedrick 1972; Borghans et al. 2004). The interruption of gene flow across fragmented populations and the reduction of effective population sizes can therefore result in a loss of genetic diversity through genetic drift and inbreeding (Wright 1969; Keller and Waller 2002; Frankham et al. 2002) and may disrupt balancing selection (Hughes and Yeager 1998) that might consequently compromise population capacity to respond to changing pathogenic pressures (O’Brien and Evermann 1988).

In support of this assumption, a possible link between highly divergent or specific MHC genotypes and individual fitness (Schad et al. 2005; Schwensow et al. 2007, 2010a, b; Sommer et al. 2014) and mate choice (Schwensow et al. 2008a, b; Huchard et al. 2013) has been suggested for two widely distributed lemurs—*Microcebus murinus* and *Cheirogaleus medius* (Cheirogaleidae, Primates). Consequently, the evaluation of MHC variation retained in populations of endangered confamiliar species can provide valuable insights to assess their viability. The complexity of MHC genotyping has long impaired detailed genetic studies of free-ranging species with unknown genomic organisation. Next-generation sequencing (NGS) technologies promise progress in this area by overcoming some of the technical difficulties associated with the complexity of MHC genotyping and by allowing cost-effective processing of large datasets (reviewed in Babik 2010; Huchard and Pechouskova 2014; Koboldt et al. 2013; Lighten et al. 2014a).

Here, we investigated MHC variation (allelic richness and divergence) and patterns of selection of two highly polymorphic MHC class II genes, DRB and DQB, in the endangered Madame Berthe’s mouse lemur, *Microcebus berthae* (B1ab.i-iii, Andriaholinirina et al. 2014), by genotyping a total of 100 individuals sampled over 9 years in three study areas. This world’s smallest primate (ca. 30 g) is endemic to the dry forests of the Menabe region in western Madagascar (Schmid and Kappeler 1994; Ganzhorn et al. 2001; Schäffler and Kappeler 2014), which has recently been identified as a priority site for conservation (Schwitzer et al. 2013a). The distribution of *M. berthae* is restricted to an area of less than 810 km^2^ within two forest fragments and a narrow corridor connecting them (e.g. Rasoloarison et al. 2000; Schäffler and Kappeler 2014). Its population density varies across its geographic range (30–100 individuals/km^2^) and seems to be affected by habitat heterogeneity and anthropogenic disturbances (Schwab and Ganzhorn 2004; Schäffler and Kappeler 2014).

Next, we compared our findings with data obtained across a similar temporal scale from a population of *M. murinus*, a sympatric congener (e.g. Weisrock et al. 2010), which in comparison to *M. berthae* presents several key ecological and demographic differences. *M. berthae* is a feeding specialist relying mostly on dispersed fast depleting resources, such as homopteran secretions or arthropods. This feeding strategy is thought to promote an intense scramble competition leading to spatial avoidance, overdispersion and lower rate of social interactions among conspecifics (Dammhahn and Kappeler 2009). In contrast, the more opportunistic feeding niche of *M. murinus*, including diverse plant and animal matter, seems to reduce competition and facilitate spatial proximity and social interactions among conspecifics (Dammhahn and Kappeler 2008a, b, 2010). The ecological flexibility of *M. murinus* is also reflected by its wide distribution across southern and western Madagascar. Its population density is higher and population size larger in Kirindy Forest than those of *M. berthae* (Eberle and Kappeler 2004; Dammhahn and Kappeler 2005, 2008b). We expect these inter-specific contrasts to influence MHC variation. First, given that allelic richness appears to be a function of effective population size (Hedrick 1985), we expect MHC allelic richness of *M. berthae* to be lower than in *M. murinus.* Second, the larger population of *M. murinus* might harbour a more diverse array of pathogens (Anderson and May 1978; Nunn et al. 2003; Hughes and Page 2007; see also Altizer et al. 2007), and this effect might be enhanced by a broader feeding niche and more frequent encounters with conspecifics offering a greater chance of pathogen encounter and transmission. In contrast, low population densities and low rates of social interactions in combination with a narrow feeding niche could result in relaxed pathogen-mediated pressure in *M. berthae*. As such, we predict to detect weaker tracks of pathogen-driven selection on *M. berthae* MHC alleles compared to *M. murinus*.

## Methods

### Sample collection and DNA extraction

DNA samples were collected from *M. berthae* from three sub-populations captured between 2005 and 2013 using baited Sherman life traps set within 25-ha study areas (N5, CS7 and Savannah) located within a 12.500-ha concession of Kirindy Forest of the Centre National de Formation, d’Etude et de Recherche en Environnement et Foresterie (CNFEREF) de Morondava (Madagascar: 44° 39′ E, 20° 03′ S, Kappeler and Fichtel 2012). The centres of the study areas N5-CS7 and Savannah-CS7 are situated ca. 2–2.5 km and N5-Savannah ca. 4–4.5 km away from each other, respectively. In total, we collected samples from 100 individuals, with sample sizes reflecting contrasting population densities and sampling effort across the years at each study area (Electronic supplementary material ESM 1; N5: *n* = 80; 42♂/37♀/1 n.a.; CS7: *n* = 14, 1♂/13 n.a.; Savannah: *n* = 6, 3♀/3♂). At first capture, each individual was briefly immobilised with 10 μl Ketanest 100 (s.c.) (Rensing 1999), individually marked with a sub-dermal microtransponder (Trovan, Usling, Germany) for other studies (e.g. Dammhahn and Kappeler 2005, 2008a, b, 2010), and a small ear biopsy of 2–3 mm^2^ was taken and preserved in 70 % ethanol. Genomic DNA was extracted from ear biopsies following standard protocol (Qiagen QIAmp DNA Mini-Kit, Qiagen Germany). We have adhered to the Guidelines for the Treatment of Animals in Behavioural Research and Teaching (Animal Behaviour 2006, 71: 245-253) and the legal requirements of the country (Madagascar) in which the fieldwork was carried out.

### 454 library preparation

PCR amplification targeting the two loci of the most variable parts of the MHC class II region, DRB and DQB, was performed using primers that flank the functionally important ABS and captured the full variability in the congener *M. murinus* (Schad et al. 2004; Averdam et al. 2011). PCR reaction mix and amplification conditions are summarised in ESM 2. Each individual PCR product (further referred to as amplicon) was electrophoresed on 1 % agarose gel to verify successful amplification. Primer design and the preparation of locus-specific amplicon libraries were described elsewhere (Huchard et al. 2012). Sequencing was conducted according to standard protocols for GS Junior sequencing (Roche, 454 pyrosequencing). All sequencing reads retrieved from a total of six sequencing runs were processed according to a post-sequencing quality control procedure following Huchard et al. (2012).

### 454 library processing

#### Allelic discrimination and evaluation of the number of loci

Artefactual alleles introduced by PCR or sequencing errors and assessing the sequencing depth necessary for reliable genotyping are well-known technical challenges associated with NGS that might compromise the reliability of assessments of MHC polymorphism (reviewed in Babik 2010; Lighten et al. 2014a).

Here, we adjusted some of the filtering steps proposed by previous authors (e.g. Babik et al. 2009; Galan et al. 2010; Zagalska-Neubauer et al. 2010; Huchard et al. 2012) to discriminate true versus artefactual alleles, relying on two central assumptions: (1) Artefactual alleles should show high similarity to one of the two parental sequences they originated from within amplicons, either by single point mutation or indels causing a shift in the reading frame, or by recombination of the two parental sequences (chimeras), and (2) artefactual alleles should be relatively rare, compared to true alleles, both across and within amplicons. In contrast to studies mentioned above, we did not identify artefactual alleles based on global allelic frequency thresholds established across amplicons but evaluated allele status both across and within each amplicon.

Both manual alignment (Multalin; Corpet 1988) and two numeric indices of allelic frequency were used to critically evaluate a potential number of loci and to discriminate true alleles from artefacts: (1) the mean per amplicon frequency (MPAF) of any given allele as the proportion of reads from an amplicon assigned to this allele, averaged across all amplicons possessing this allele, and (2) the relative per amplicon frequency (RPAF) of each allele as the proportion of reads retrieved for each given allele within a given amplicon. We predicted that artefactual alleles should be relatively rare, compared to true alleles across amplicons (reflected by low MPAF) and within amplicons (reflected by low RPAF). While MPAF can help to identify artefacts returned at low frequencies across sequencing runs, RPAF can help to identify heterogeneities in the distribution of within-amplicon allelic frequency. These can be either artefactual alleles that occur non-randomly across sequencing runs or at high frequencies only in few amplicons, skewing their MPAF (i.e. run-specific sequencing errors or homopolymers) (see also Lenz and Becker 2008; Harismendy et al. 2009; Gilles et al. 2011; Sommer et al. 2013; Lighten et al. 2014a, b), or cross-amplicon contaminations of true alleles (with high MPAF and low RPAF). The occurrence of such DNA carryover contaminants has recently been acknowledged as an underrated source of genotyping errors associated with NGS (Huchard et al. 2012; Li and Stoneking 2012; Sommer et al. 2013; Lighten et al. 2014a, b). Here, we did not systematically eliminate these suspected contaminants by using a fixed threshold for a minimum frequency per amplicon (here referred to as RPAF) under which alleles are filtered out within amplicons (e.g. Sepil et al. 2012; Huchard et al. 2012), to avoid eliminating true alleles and thereby generating potential allelic dropout (van Oosterhout et al. 2006; Sommer et al. 2013). Rather, the status of amplicons suspected of contaminations by true alleles was clarified by replicating affected amplicons.

In the first step of the allele sorting procedure, we attempted to evaluate whether target genes may be duplicated in order to assess the sequencing depth required to ensure reliable genotyping. Although there was no indication of a loci duplication in *M. murinus* (Averdam et al. 2011; Huchard et al. 2012), we could not assume the same in *M. berthae* due to extensive variation in the genomic organisation of MHC within and across species (e.g. Kelley et al. 2005; Winternitz and Wares 2013; Lighten et al. 2014b). Therefore, we investigated the MPAF and RPAF distribution of the most common to the least common alleles across all amplicons, assuming that in the case of non-duplication, we would detect a notable drop between the two most common and remaining alleles (see also Babik et al. 2009; Huchard et al. 2012). Next, we evaluated our findings by manual alignment of all alleles within each amplicon and attempted to discriminate artefactual from true alleles based on our assumptions—similarity to parental allele and low MPAF and RPAF. When the status of alleles remained ambiguous, affected amplicons were replicated. Finally, we replicated those few amplicons that passed allele sorting with more true alleles than expected given the estimated number of loci to check whether this came from the genuine locus duplication or from an artefact.

#### Assessment of minimum sequencing depth and genotyping reliability

Based on the estimated number of loci, we proceeded with a final screening step to evaluate the sequencing depth necessary for accurate genotyping using the program ‘Negative Multinomial’ (http://www.lirmm.fr/~caraux/Bioinformatics/NegativeMultinomial/) developed by Galan et al. (2010). Amplicons that did not return a sufficient amount of reads based on this estimate were re-genotyped.

The efficiency of allele-sorting was then evaluated through two steps. First, assuming that artefacts originated during a single PCR reaction or introduced by a genotyping mistake would not occur independently in many amplicons, we investigated the relationship between the MPAF of each allele and the number of amplicons possessing this allele. We then correlated both values before and after allele sorting. Here, we expected a positive relationship before allele sorting—due to the fact that artefacts should display both low MPAF and be retrieved in few individuals only—that would disappear after allele sorting (Babik et al. 2009; Huchard et al. 2012). Second, a set of amplicons were additionally replicated for each loci within independent sequencing runs (DRB *n* = 23; DQB *n* = 27) to assess the reliability of our genotyping for each locus within.

### Sequence analysis and phylogeny reconstruction

All true alleles were retained for downstream analysis, including those that were possessed by one individual only to prevent the elimination of rare alleles leading to an underestimation of allelic richness and to avoid generating false homozygotes. However, these alleles were not submitted in public repositories.

Allelic divergence was evaluated by computing average pairwise distances (number of differences) among all pairs of nucleotide and amino acid sequences in each locus in MEGA 6 (Tamura et al. 2013). Evolutionary relationships between amino acid sequences of both loci found by this study in *M. berthae* and previously in *M. murinus* were constructed in MEGA, using a neighbour-joining algorithm with Poisson correction (Saitou and Nei 1987; Zuckerkandl and Pauling 1965). *Mimu* sequences originated from 664 individuals captured within the study area CS7 between the years 2000 and 2010. These *Mimu* alleles were retrieved using 454 technology and comparable allele validation steps (see Huchard et al. 2012). The repeatability of sequence alignment was determined by a bootstrap analysis with 1000 replications.

Allelic richness of both loci detected within the study population of *M. berthae* was compared to those previously described for *M. murinus* (Huchard et al. 2012). To evaluate the number of alleles detected for a given sampling effort, we conducted a permutation test in R (www.r-project.org). Here, we randomly selected 10 individuals and counted the number of distinct alleles detected. This procedure was repeated 100 times to calculate a mean and SD for each sampling effort, adding 10 individuals at each step until 100. This procedure was conducted for both *M. berthae* and *M. murinus* and plotted for both loci to illustrate the number of distinct alleles detected per given sampling effort.

### Population genetic analysis

Linkage disequilibrium between DRB and DQB loci was tested using a likelihood ratio test, where the null hypothesis of no association between loci (linkage equilibrium) is compared to the hypothesis of a possible association (Slatkin and Excoffier 1996). The significance of the procedure is found by computing the null distribution of this ratio under the hypothesis of linkage equilibrium using 10,000 permutations implemented in Arlequin 3.5.1.3. (Excoffier and Lischer 2010). Using GENEPOP v 1.2 (Rousset and Raymond 1995), a null allelic frequency was estimated by maximum likelihood estimation (EM algorithm; Dempster et al. 1977), and deviations from Hardy-Weinberg equilibrium (HWE) were calculated for each locus separately using the exact *U*-score test (Rousset and Raymond 1995), with the alternative hypothesis predicting heterozygote excess. The extent of genetic differentiation among sub-populations (regardless of sex and year cohort) was examined by pairwise *F*_ST_ at both loci using Arlequin (10,000 permutations, Wright 1965). Comparisons between adult males and females and across year cohorts were not included due to uneven and small sample sizes (see above and ESM 1).

### Test of positive selection

The presence of positively selected sites (PSS) was investigated in both genes separately. PSS are characterised by *ω* > 1 with *ω* = *d*_*N*_/*d*_*S*_, and *d*_*N*_ and *d*_*S*_ being the relative amounts of substitutions at non-silent (*d*_*N*_) and silent (*d*_*S*_) codon sites. First, we investigated the strength of positive selection using the likelihood ratio modelling approach. We compared two models of the codon evolution: the null model, where *ω* < 1 and varies according to the beta distribution (model M7), and a model allowing an additional class of sites, where *ω* > 1, to account for a possible occurrence of PSS (model M8) using a likelihood ratio test (LRT) (in Yang et al. 2000). If model M8 fits the data better than M7, PSS were identified through Bayes Empirical Bayes (BEB) procedure and retained for evaluation by the next step if statistically significant (Yang et al. 2005), using the package CodeML implemented in PAML 4.7 (Yang 2007).

As a second approach, we estimated values of *d*_*N*_ and *d*_*S*_ and their standard errors by calculating the pairwise number of silent and non-silent substitutions (Nei and Gojobori 1986) applying Jukes-Cantor correction for multiple hits implemented in MEGA. This rather conservative approach considers all possible evolutionary pathways (excluding termination codons) leading from one codon to another as equally probable and is thereby expected to provide conservative (minimum) estimates of numbers of substitutions compared to the positive selection hypothesis *ω* > 1 (Nei and Kumar 2000). A codon-based *Z*-test of selection was performed to test whether both PSS (*ω* > 1) and non-PSS (*ω* < 1), identified by the previous approach using BEB procedure, were under positive selection. Furthermore, we compared *ω* in ABS and non-ABS. Their location was derived from referential human sequences (HLA-DQB, HLA-DRB; Bondinas et al. 2007) and compared with previously detected PSS and non-PSS. Finally, overall values of *d*_*N*_ and *d*_*S*_ were calculated.

## Results

### Allelic discrimination and evaluation of the number of loci

A total of 321 unique sequences were retrieved for DRB from 148 amplicons (including 49 replicates, see below) and 105 sequences for DQB from 130 amplicons (including 32 replicates), in the range of 162–170 bp (excluding primers). For more details and statistics of sequencing outcome, see ESM 3. The distribution of MPAF and RPAF from the first to sixth most common alleles averaged across all amplicons revealed a notable drop of frequency between the two most common and remaining alleles, suggesting no duplication of either locus (ESM 4).

Within 321 DRB sequences, 286 (89 %) displayed MPAF < 0.05 and were identified by manual alignment as artefacts (95 %) or contaminants (5 %), following our criteria. From the remaining 35 (11 %) alleles with MPAF ≥ 0.05, 13 alleles were identified as artefacts. Additionally, five alleles occurring within a single amplicon (MPAF 0.05–0.08) were identified as contaminants and discarded (see below). The remaining 17 alleles (MPAF 0.08–0.69) were retained as true alleles. In DQB, 74 (70 %) of all 105 sequences displayed MPAF < 0.05, and all of those were eliminated as artefacts (95 %) or contaminants (5 %). Among 31 remaining sequences with MPAF ≥ 0.05, four more sequences were eliminated as artefacts and five as contaminants (MPAF 0.06–0.24). The remaining 22 sequences were retained as true alleles. In both loci, artefacts with MPAF ≥ 0.05 mostly represented homopolymer indels—occurring either at inconsistent frequencies across sequencing runs, within a single sequencing run or amplicon—and their elimination was further supported by running replicates of affected amplicons. Nine out of 14 contaminants were eliminated by running replicates and three in DRB and two in DQB occurred within one amplicon that was due to extensive inflation of artefacts, and thereby inconclusive genotype, excluded in each locus. Overall, 304 (95 %) sequences in DRB and 83 (79 %) in DQB were eliminated. After allele sorting, only three (out of 96 individuals) and six (out of 98 individuals) amplicons had more than two true alleles in DRB and DQB, respectively. In all of them, the existence of a third and in one case fourth allele could be excluded by re-sequencing affected amplicons, which further confirmed our assumption that both loci were non-duplicated.

### Assessment of minimum sequencing depth and genotyping reliability

Based on our conclusions of no loci duplication, we used the probabilistic model (Galan et al. 2010) and estimated that a minimum of 18 reads were required for reliable genotyping of each given locus with a confidence level of 0.95. All amplicons with <18 reads (DRB 26; DQB 5) were re-genotyped and replaced in the dataset, except of three amplicons in DRB that did not return >18 reads in the second genotyping attempt. Additionally, 23 amplicons for DRB and 27 for DQB were genotyped in replicates to estimate genotyping reliability, and all of them showed a perfect reproducibility of assigned genotypes.

The correlation between the MPAF of each allele and the number of amplicons possessing this allele was significant before allele sorting in both loci (Pearson’s correlation, DRB *n* = 321 alleles, *r* = 0.78, *P* < 10^−15^; DQB *n* = 105 alleles, *r* = 0.69, *P* < 10^−15^), largely driven by the presence of alleles that had both low MPAF and low frequency (ESM 5). This correlation disappeared in DQB after discarding artefacts (Pearson’s correlation *n* = 22 alleles, *r* = 0.22, *P* = 0.33) and was weakened in DRB, though still significant (Pearson’s correlation, DRB *n* = 17 alleles, *r* = 0.64, *P* < 0.006).

### Sequence analysis and phylogeny reconstruction

From the total of 17 *Mibe*-DRB and 22 *Mibe*-DQB sequences found in 96 and 98 individuals of *M. berthae*, respectively, none have been described previously. Accession numbers of these alleles as well as the full nucleotide sequence of alleles occurring in only one individual are given in Appendix (Table 3).

Average nucleotide divergence (number of differences) between sequences was comparably high in both loci (mean ± SD; DRB 17.07 ± 2.44; DQB 14.44 ± 2.16). Among *Mibe*-DRB sequences, we found 47 (29 %) variable nucleotide sites and 35 (21 %) among *Mibe*-DQB sequences. Each nucleotide sequence of both loci translated into a unique amino acid sequence and the absence of stop codons suggests that all sequences can encode functional proteins. Amino acid sequences revealed 23 (43 %; DRB) and 23 (41 %; DQB) variable sites out of 54 and 56 sites (see below), and comparable amino acid divergence was found in both loci (mean ± SD; DRB 11.73 ± 2.0, DQB 10.76 ± 1.82).

**Fig. 1:**
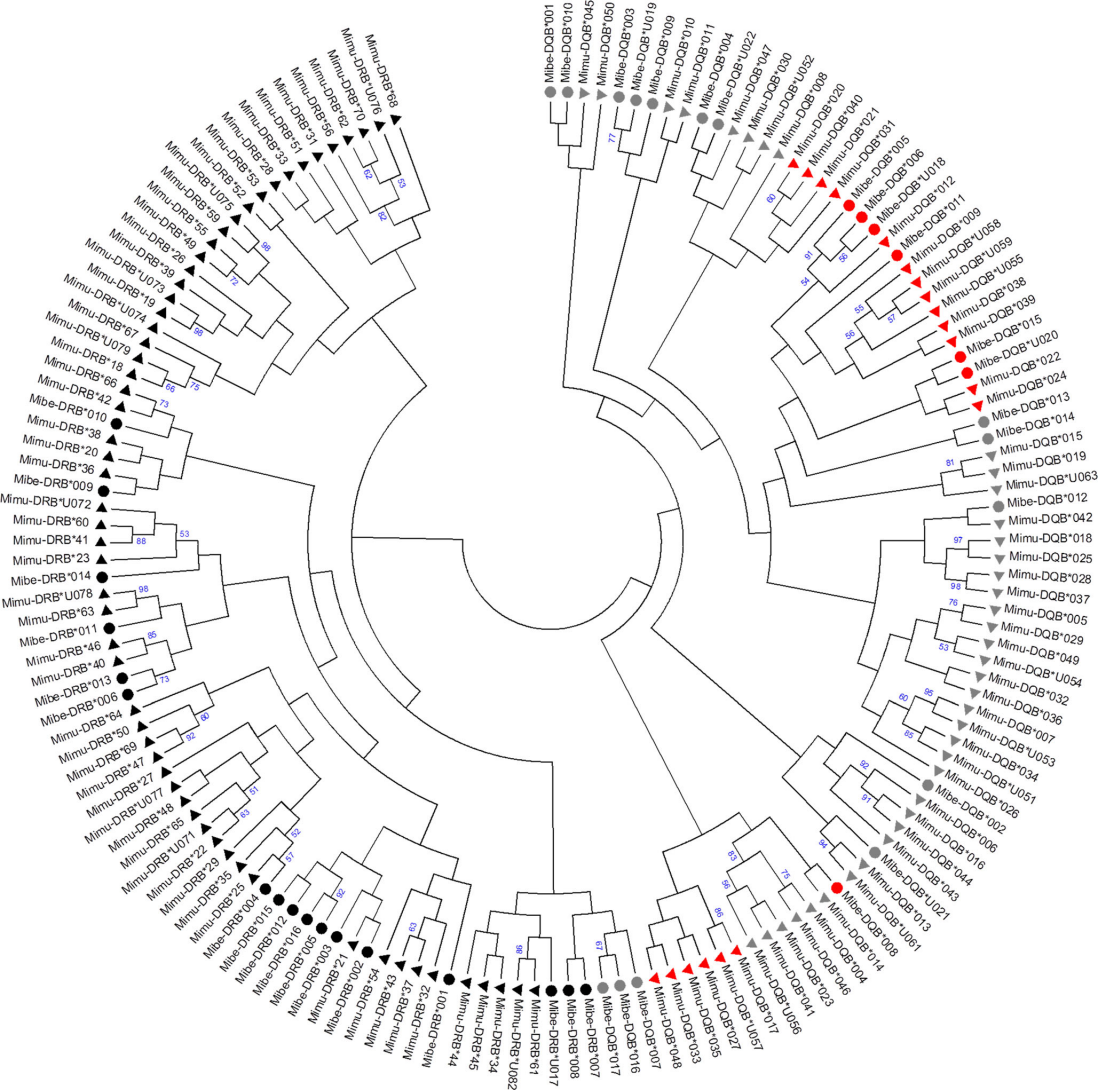
Evolutionary relationships between amino acid sequences for 17 *Mibe*-DRB (*black circles*) and 22 *Mibe*-DQB sequences (*grey circles* for sequences without 6-bp insertion and *red circles* for sequences with the insertion) described in this study, including 59 *Mimu*-DRB (*black triangles*) and 58 *Mimu*-DQB sequences (*grey triangles* for sequences without two-codon insertion and *red triangles* for sequences with it) described in Huchard et al. (2012). The tree configuration was derived using neighbour-joining algorithm (Bootstrap 1000; Poisson correction) in MEGA 6. Only bootstrap values exceeding 50 % are shown. Accession numbers and nucleotide sequences of *M. berthae* are presented in Appendix (Table 3) (colour figure online)

The reconstruction of evolutionary relationships between amino acid sequences of both species revealed two distinct loci-specific clusters, with the exception of three *Mibe*-DQB sequences (*Mibe-*DQB*017, *016 and *007), that clustered separately among DRB sequences of both *M. berthae* and *M. murinus* (Fig. 1). These three *Mibe* sequences were retrieved independently at least in two and up to 11 individual amplicons across different sequencing runs, and their presence and loci identity were confirmed by replicating from one to three amplicons possessing affected sequences. Moreover, these three sequences clustered with other DQB sequences when including only *Mibe* sequences in the analysis (data not shown). There was no clear separation between sequences of the two species in either locus (Fig. 1), and the level of amino acid divergence was comparable within (see above and Huchard et al. 2012) and between allelic pools of the two species (*Mimu* vs. *Mibe*-DRB 11.6 ± 2.1; DQB 11.3 ± 1.9). Additionally, an insertion (two codons) causing fragment length polymorphism in DQB, homologous to the one previously described in 19 *Mimu*-DQB sequences (Huchard et al. 2012), was detected in seven *Mibe*-DQB sequences. This insertion did not result in a shift of the reading frame, and there was no evidence for stop codons that would indicate a loss of function. These sequences created large distinct clusters, except for one *Mibe* sequence (*Mibe*-DQB*008, Fig. 1).

**Fig. 2:**
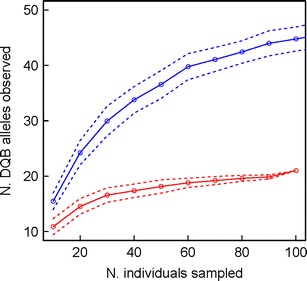
Estimation of allelic richness for a given sampling effort through re-sampling procedure, showing the number of distinct alleles detected when randomly drawing an increasing number of individuals from our sample in *M. berthae* (*red*) and *M. murinus* (*blue*). Given the similarity of the observed pattern between DRB and DQB, only the plot for DQB loci is shown. The *dotted lines* indicate the standard deviation around the estimated mean (*solid line*) (colour figure online)

Allelic richness (number of distinct alleles) estimated for a given sampling effort (number of sampled individuals) through re-sampling procedure in *M. berthae* and *M. murinus* is shown in Fig. 2. The estimated mean of distinct alleles detected per sampling effort is lower in *M. berthae* in both loci, indicating lower overall allelic richness for a given sampling effort in this study population.

### Population genetic analysis

**Fig. 3:**
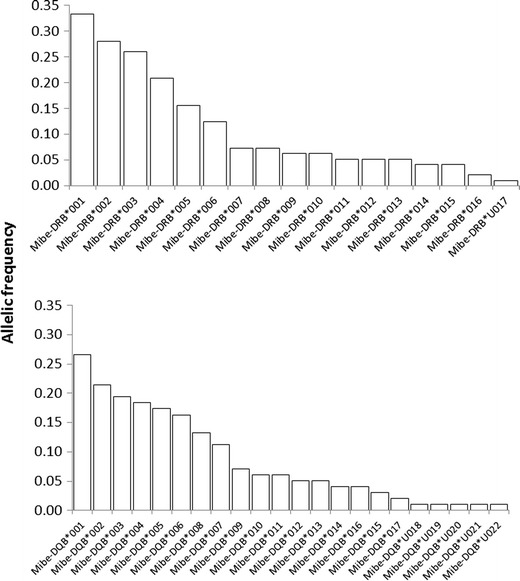
The distribution of allelic frequencies (i.e., rate of occurrence) of 17 MHC-DRB and 22 MHC-DQB alleles within the study population of *M. berthae* (DRB, *n*_ind_ = 96; DQB, *n*_ind_ = 98)

The null hypothesis of linkage equilibrium between loci could be rejected (*χ* ^2^ = 810.33, *df* = 396, *P* < 10^−06^). Allelic distribution patterns were relatively similar in both genes across all sub-populations with allelic frequencies varying widely within each locus from 1 to 33 % in DRB and 1 to 27 % in DQB (Fig. 3).

The estimated frequency of null alleles was low in both loci (<0.01). The comparable observed and expected level of heterozygosity could be found at both loci across all sub-populations (DRB, *H*_O_ = 0.91, *H*_E_ = 0.90; DQB, *H*_O_ and *H*_E_ = 0.92), and heterozygote excess was not detected in either locus (DRB, Fis^W^&^C^ = −0.007, *P* = 0.58; DQB, Fis^W^&^C^ = 0.005, *P* = 0.80).

Pairwise comparisons did not reveal any genetic differentiation in either loci among the three sub-populations (for all pairs, *F*_ST_ < 0.001; *P =* 0.49-0.84), suggesting intact/ongoing gene flow among them. The allelic frequencies within each sub-population and across year cohorts of the largest sub-population (N5) are shown in ESM 6.

### Test of positive selection

**Fig. 4:**
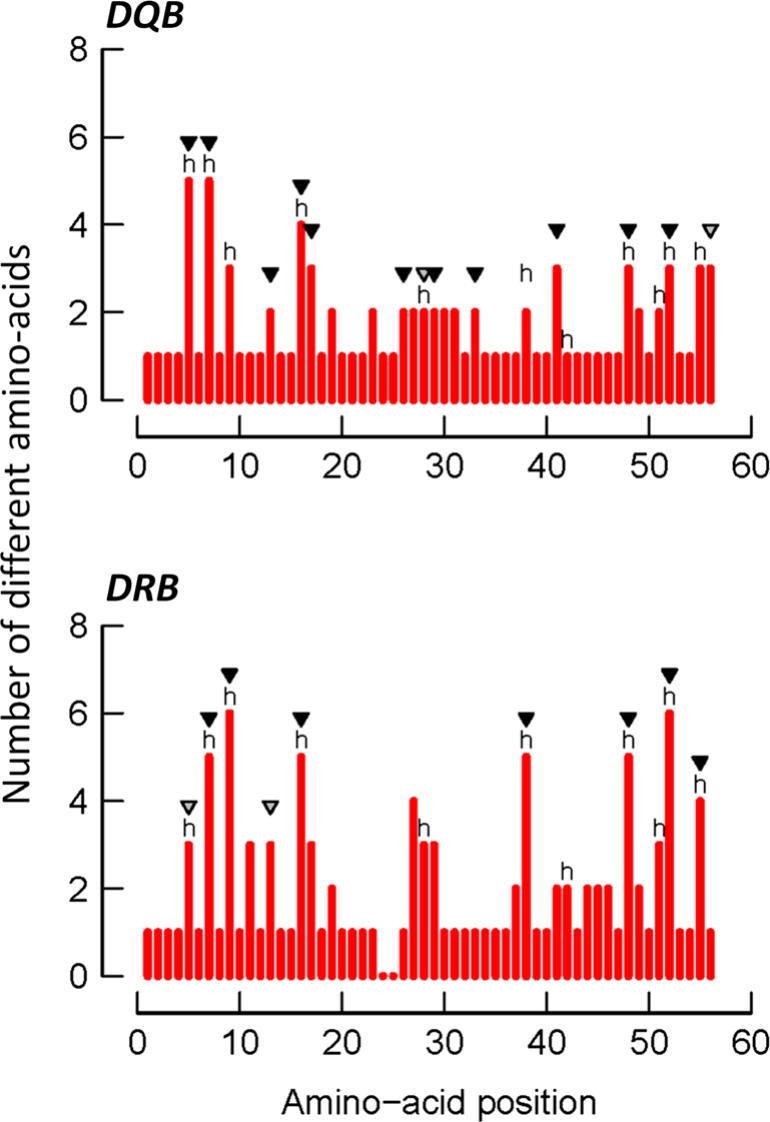
Amino acid variation plots for *Mibe*-DQB and *Mibe*-DRB alleles. Human antigen-binding sites (ABS) are indicated with the letter *h* (Bondinas et al. 2007), and positively selected sites (PSS) are indicated by *black* (*P* > 99 %) and *grey triangles* (*P* > 95 %). The insertion of two codons at positions 24–25 in seven DQB alleles causes a gap in sequences of DRB loci

The significant deviation of the LRT statistics from a *χ* ^2^ distribution allowed rejection of the null model assuming neutral evolution (M7) in favour of a model allowing for a class of sites being subjected to diversifying selection (M8) for both loci (Table 1). In DRB, nine PSS were identified (CI 99 %, *n* = 7; CI 95 %, *n* = 2). Eight of those occurred at homologous positions with HLA-ABS, and another one was located within a three amino acid distance (Fig. 4b). In DQB, 13 PSS were detected (CI 99 %, *n* = 11; CI 95 %, *n* = 2). Six out of 13 PSS were homologous to HLA-ABS, seven other located within one to four amino acid distance (Fig. 4a). In comparison, two out of three DQB-PSS described in *M. murinus* (Huchard et al. 2012) were homologous to those identified in *M. berthae* (positions 5 and 16). Additionally, six PSS were homologous between the two species in DRB.

**Table 1:** Evaluation of the goodness of fit for different models of codon evolution and estimated parameter values

Model	LnL^a^	Kappa (ts/tv)	AIC	∆AIC^b^	Parameters
MHC-DRB					
M0—one *ω*	−922.27	0.79	1846.12	184.38	*ω* ^c^ = 0.61
M7—nearly neutral with *β* ^g^	−839.49	0.86	1680.7	18.96	
M8—positive selection with *β* (*ω*0 ≤ 1, *ω*1 > 1)^h^	−830.14	0.73	1661.74	Best	*p*0^d^ = 0.78, *p*1^e^ = 0.22 , *ω* ^f^ = 3.01
MHC-DQB					
M0—one *ω*	−873.89	1.34	1750.45	150.84	*ω* = 1.29
M7—nearly neutral with *β*	−810.82	1.66	1624.97	25.36	
M8—positive selection with *β* (*ω*0 ≤ 1, *ω*1 > 1)	−798.3	1.5	1599.61	Best	*p*0 = 0.73, *p*1 = 0.27, *ω* = 4.24

*AIC* Akaike information criterion, *Kappa (ts/tv)* transition/transversion rate

^a^Log likelihood of a model

^b^Difference between the value of the AIC of a given model and the best model

^c^ *d*_*N*_/*d*_*S*_

^d^Proportion of sites with *ω* ≤ 1

^e^Proportion of positively selected sites (*ω* > 1)

^f^Estimated value of *ω* for sites under positive selection

^g^For all sites, *ω* ≤ 1 and the *β* distribution approximates *ω* variation

^h^A proportion of sites evolves with *ω* > 1

The estimated values of *d*_*N*_ and *d*_*S*_ (±SE) through evolutionary pathways method (Table 2) confirmed the results of the first analysis by revealing an elevated *d*_*N*_ relative to *d*_*S*_ in all ABS versus non-ABS and in all PSS versus non-PSS. Codon-based *Z*-tests of selection indicated that all PSS and ABS have been affected by positive selection in both loci.

**Table 2:** Results of evolutionary pathway method (MEGA 6) to estimate values of *d*_*N*_ and *d*_*S*_ (±SE) for ABS and non-ABS defined by homology with HLA and for PSS and non-PSS identified by Bayes Empirical Bayes (BEB) analysis (PAML)

Positions	Number of codons in each category	*d*_*N*_	*d*_*S*_	*Z*	*P*
MHC-DRB					
ABS	11	0.59 ± 0.09	0.05 ± 0.04	5.038	0.000
Non-ABS	43	0.05 ± 0.02	0.06 ± 0.03	-0.470	0.639
PSS	9	0.75 ± 0.08	0.17 ± 0.14	3.226	0.002
Non-PSS	45	0.06 ± 0.02	0.04 ± 0.02	0.484	0.629
All	54	0.13 ± 0.03	0.06 ± 0.03	1.921	0.057
MHC-DQB					
ABS	11	0.35 ± 0.11	0.01 ± 0.01	3.123	0.002
Non-ABS	43	0.07 ± 0.02	0.04 ± 0.02	1.493	0.138
PSS	13	0.50 ± 0.09	0.04 ± 0.04	4.912	0.000
Non-PSS	41	0.04 ± 0.01	0.03 ± 0.02	0.783	0.435
All	54	0.12 ± 0.03	0.03 ± 0.02	3.174	0.002

From Nei and Gojobori 1986, Bondinas et al. 2007, and Yang et al. 2005. *P* probability of *d*_*N*_ = *d*_*S*_ using *Z*-test of selection. All positions containing gaps and missing data were eliminated

## Discussion

This is a first description of the polymorphism of two MHC class II genes in the endangered Madame Berthe’s mouse lemur. We detected a total of 17 *Mibe*-DRB and 22 *Mibe*-DQB unique sequences which showed high divergence and tracks of past positive selection. Below, we compare patterns of variation and selection in *M. berthae* with those previously described for *M. murinus*, a closely related sympatric congener, that differs in several key aspects of its demography and ecology and discuss potential implications of these results on population viability.

### MHC variation and selection patterns

A total of 17 *Mibe*-DRB and 22 *Mibe*-DQB unique sequences were detected within members of the three sub-populations of *M. berthae* in Kirindy Forest, Western Madagascar. Comparison with 59 DRB and 58 DQB sequences of *M. murinus* obtained previously from the same study site (Huchard et al. 2012) revealed no clear separation of amino acid sequences between the two species in either locus (Fig. 1). Nucleotide and amino acid sequence similarity between alleles of the two species, as well as the presence of a two-codon insertion located at the same position in 19 *Mimu*- and 7 *Mibe*-DQB sequences, could indicate the retention of MHC motifs in both loci during periods of time exceeding the evolutionary split between species (trans-species polymorphism; Klein 1987). MHC sequence similarity limited to exons encoding peptide binding regions have been detected many times between species that were sometimes distantly related (summarised in Klein et al. 2007; Lenz et al. 2013) and may represent examples of trans-species polymorphism or result from convergent evolution (independent evolution of similar traits in response to similar ecological pressures) (e.g. Klein et al. 2007). In addition, the reconstruction of evolutionary relationships between amino acid sequences of the two loci revealed a partial paraphyly, with three Mibe-DQB sequences clustering within DRB sequences. A BLAST search of the affected *Mibe*-DQB nucleotide sequences revealed *Mimu*-DQB sequences as a close match. Moreover, when analysed separately, *Mibe* sequences generated two distinct loci-specific clusters (data not shown) supporting their correct assignment to DQB loci. Additionally, these sequences occurred within multiple individuals and their presence was confirmed by replication, which excluded the possibility of sequencing run-specific amplification mismatch. Such paraphyly has been also reported previously in *M. murinus* by Huchard et al. (2012) and may result from a combined effect of tight physical linkage, shared origin and high functional similarity between the two loci.

In contrast to 17 *Mibe*-DRB and 22 *Mibe-*DQB sequences obtained by this study from the total of ca. 100 individuals sampled over 9 years in three study areas, 59 *Mimu*-DRB and 58 *Mimu*-DQB originated from 664 individuals of *M. murinus* sampled over a comparable period of time within a single study area (Huchard et al. 2012). Allelic richness (number of distinct alleles) for a given sampling effort (number of sampled individuals) that was estimated through re-sampling procedure in dataset of both species revealed the average number of distinct alleles detected for a given sampling effort to be ca. twofold lower in *M. berthae* compared to *M. murinus* (Fig. 2). This finding indicated lower allelic richness in *M. berthae*, where sampling of ca. 60 individuals, compared to ca. 200 individuals in *M. murinus*, would allow to capture most alleles present in the study population, deduced from an inflection in the graph illustrating the relationship between allelic richness and sampling effort (see Fig. 2 and Huchard et al. 2012). Moreover, the allelic distribution across year cohorts within the largest sub-population of *M. berthae* (N5) suggests that reported allelic richness within this study site may be overestimated, since some alleles were detected exclusively within earlier cohorts and seem to have disappeared within current generations (e.g. after 2008–2009) (ESM 6). However, sample size is too small to interpret apparent fluctuations in allelic frequencies that might be further enhanced by a progressive displacement of the *M. berthae* population, located at the periphery of the study area (Dammhahn and Kappeler 2008b), or by individual migrations. The lower allelic richness found in *M. berthae* matched our predictions based on its overall lower population densities and population size relative to *M. murinus* (Dammhahn and Kappeler 2005, 2008b). Even though the reasons for lower population density of *M. berthae* are unknown, factors such as a narrow feeding niche promoting intense intra-specific scramble competition, larger home ranges and less cohesive social networks in *M. berthae* (Dammhahn and Kappeler 2005, 2008a, 2009, 2010), when compared to spatially more clumped generalist *M. murinus* (e.g. Eberle and Kappeler 2004), are thought to contribute to the naturally lower population densities in this species (Dammhahn and Kappeler 2008b). Additionally, we observed a notable decrease in the number of newly captured individuals across the years in the most densely populated study area (N5) despite of a comparable capture effort across years (ESM 1). This pattern could either be the result of a decreasing population size or, alternatively, a spatial exclusion from the study area by its superior competitor (Dammhahn and Kappeler 2008b), whose sub-population is shifting in recent years into the areas previously exclusively occupied by *M. berthae* (data not shown).

Finally, the small population size, specialist diet and lower rate of social interactions among conspecifics of *M. berthae* could to some extent promote a limited array of pathogens and its transmission across conspecifics (reviewed in Edwards and Potts 1996; Nunn et al. 2003; Vitone et al. 2004; Rifkin et al. 2012). This could in turn result in relaxed selection, possibly manifested not only by lower allelic richness and/or divergence but also by less tracks of selection on MHC sequences. In *M. berthae*, allelic divergence in both loci, as well as strong evidence of past historical balancing selection on MHC sequences (Tables 1 and 2; Fig. 4), are comparable to patterns described in *M. murinus* (Huchard et al. 2012) and do not support the idea of a weaker pathogen-driven selection in *M. berthae* compared to *M. murinus*. Thus, population size rather than weak selection seems to constrain allelic richness in this population.

In addition, nine PSS were detected across 17 *Mibe*-DRB sequences and 13 across 22 *Mibe*-DQB sequences, suggesting that DQB may be of equal or higher functional importance than DRB in this species. This contrasts with previous findings in *M. murinus*, where DRB was suggested to be under stronger diversifying selection than DQB based on their relative number of PSS (11 vs. 3) (Huchard et al. 2012). Contrasting selection patterns could reflect divergent functions of this locus in *M. berthae* versus *M. murinus*. Under this scenario, we may also expect different levels of allelic variation (richness and divergence) between the two loci. This is the case in neither *M. murinus* nor *M. berthae*. This may be due to the fact that (i) selection pressures acting on both loci are not independent given their tight linkage, (ii) allelic variation reflects variation in demography and not simply selection, or (iii) allelic variation and signatures of past positive selection reflect the strength of selection over different time scales. An elevated rate of non-synonymous mutations requires a long time to accumulate (Bryja et al. 2007) as well as to vanish after the disappearance of selection (Garrigan and Hedrick 2003), whereas fluctuations in allelic variation may be more dynamic.

### Implications for population resistance

To assess the adaptive significance and fitness consequences of MHC variation, it is essential to distinguish the relative importance of different measures of MHC polymorphism (Garamszegi and Nunn 2011). The high level of amino acid divergence among *Mibe*-alleles or their effective combination within individual genotypes may buffer potential detrimental effects of lower allelic richness for pathogen resistance. Thus, persistence of certain *Mibe*- alleles across several generations and study areas (ESM 6) could be facilitated by their divergence (i.e. divergent allele advantage hypothesis) (Richman et al. 2001; Schwensow et al. 2010b; Lenz et al. 2009; Lenz 2011; Froeschke and Sommer 2012; Sepil et al. 2013) or by the effect of MHC-dependent mate choice favouring specific alleles conferring resistance against dominant pathogens (e.g. Hill et al. 1991; Schad et al. 2005; Schwensow et al. 2007, 2010a; Axtner and Sommer 2012; Kloch et al. 2013). However, high allelic divergence may not be sufficient to maintain effective flexibility of the immune response in the long-term when allelic richness is low. In small populations with limited gene flow, genetic drift may weaken the capability of balancing selection to maintain high levels of MHC polymorphism through disappearance of rare allelic variants (Hartl and Clark 1997; Ejsmond and Radwan 2011). This might in turn compromise the capability of the host’s immune system to keep up with the evasive mechanisms of the current, or newly introduced pathogens. However, whether and how MHC variation found in *M. berthae* translates into population viability remains to be tested by the integration of genetic data with further health and survival assessment.

Overall, the empirical evidence supporting a link between MHC variation and fitness remains equivocal across taxa (reviewed in Acevedo-Whitehouse and Cunningham 2006; Radwan et al. 2010; Winternitz et al. 2013). Some populations that have undergone a demographic bottleneck seem to cope with critically low MHC variation (e.g. Ellegren et al. 1993; Mikko and Andersson 1995; Babik et al. 2005; Gangoso et al. 2012), or low MHC allelic richness compensated by high allelic divergence (e.g. Radwan et al. 2007; Castro-Prieto et al. 2011), while others retained high levels of MHC variation despite facing a bottleneck that simultaneously lowered neutral genetic diversity (Aguilar et al. 2004; Hedrick and Hurt 2012; Oliver and Piertney 2012). Although the consequences of decreasing MHC variation might be undetectable over long periods of time, it might eventually compromise the ability of small or isolated populations to resist to ever-changing pathogen pressures in the future, over time scales that may be difficult to measure in most empirical studies (reviewed in Radwan et al. 2010; Spurgin and Richardson 2010). Therefore, the continuous long-term demographic monitoring of populations for which estimates of MHC variation have been established at one or several points in time may, in the future, help us to refine our understanding of the time scale over which such processes are acting, especially in relatively short-lived species.

## Electronic supplementary material

Below is the link to the electronic supplementary material.

ESM 1 (DOCX 79 kb)

ESM 2 (DOCX 14 kb)

ESM 3 (DOCX 12 kb)

ESM 4 (DOCX 172 kb)

ESM 5 (DOCX 124 kb)

ESM 6 (DOCX 22 kb)

## Appendix

**Table 3:** A List and accession numbers of *Mibe*-DRB and *Mibe*-DQB sequences described by this study

Allele name	Number of individuals	Length (bp)	GenBank accession number/nucleotide sequence (5′–3′)
*Mibe*-DRB*001	33	163	LN610539
*Mibe*-DRB*002	27	163	LN610540
*Mibe*-DRB*003	25	163	LN610541
*Mibe*-DRB*004	21	163	LN610542
*Mibe*-DRB*005	17	163	LN610543
*Mibe*-DRB*006	11	163	LN610544
*Mibe*-DRB*007	7	163	LN610545
*Mibe*-DRB*008	6	163	LN610546
*Mibe*-DRB*009	6	163	LN610547
*Mibe*-DRB*010	6	163	LN610548
*Mibe*-DRB*011	5	163	LN610549
*Mibe*-DRB*012	5	163	LN610550
*Mibe*-DRB*013	5	163	LN610551
*Mibe*-DRB*014	4	163	LN610552
*Mibe*-DRB*015	4	163	LN610553
*Mibe*-DRB*016	2	163	LN610554
*Mibe*-DRB*U017	1	163	CAGCGGGTGCGGCTCCTGGTGAGAGGCATCTACAACCGCGAGGAGTTCCTGCGCTACGACAGCGACGTGG
			GCAAGTACCGGGCGGTGACGGAGCTGGGCCGGCCGGACGCCGAGTCCTTGAACCGCCAGCAGGACCACCT
			GGAGCAGAGGCGGGCCGCGGTGG
*Mibe*-DQB*001	25	163	LN610555
*Mibe*-DQB*002	20	163	LN610556
*Mibe*-DQB*003	19	163	LN610557
*Mibe*-DQB*004	18	163	LN610558
*Mibe*-DQB*005	17	169	LN610559
*Mibe*-DQB*006	16	169	LN610560
*Mibe*-DQB*007	13	163	LN610561
*Mibe*-DQB*008	11	169	LN610562
*Mibe*-DQB*009	7	163	LN610563
*Mibe*-DQB*010	6	163	LN610564
*Mibe*-DQB*011	6	169	LN610565
*Mibe*-DQB*012	5	163	LN610566
*Mibe*-DQB*013	5	163	LN610567
*Mibe*-DQB*014	5	163	LN610568
*Mibe*-DQB*015	3	169	LN610569
*Mibe*-DQB*016	3	163	LN610570
*Mibe*-DQB*017	2	163	LN610571
*Mibe*-DQB*U018	1	169	CAGCGGGTGCGGAGTGTGAACAGATACATCTACAACCAGGAGGAGTTCGTGCGCTTCGACAGCGACATCG
			GCTTGGGCGAGTACCTGGCGGTGACGGAGCTGGGCCGGCCGGAGGCCGAGCACTGGAACCGCCAGCAGGA
			CCTCCTGGAGCAGAAGCGGGCCGCGGTGG
*Mibe*-DQB*U019	1	163	CAGCGGGTGCGGTATGTGACCAGATACATCTACAACCGCGAGGAGACCGTGCGCTTCGACAGCGACGTGG
			GCGAGTACCTGGCCATGACGCCGCTGGGCCGGCCGGACGCCGAGTACTGGAACCGCCAGCAGGACATCCT
			GGAGCAGACGCGGGCCGAGCTGG
*Mibe*-DQB*U020	1	169	CAGCGGGTGCGGCTTGTGACCAGATACATCTACAACCAGGAGGAGTTCGTGCGCTTCGACAGCGACATCG
			GCTTGGGCGAGTACCGGGCCGTGACGGAGCTGGGCCGGCCGGACGCCGAGTCCTGGAACCGCCAGCAGGA
			CTTCATGGAGCAGAGGCGGGCCGAGGTGG
*Mibe*-DQB*U021	1	163	CAGCGGGTGCGGCATGTGGTCAGACACATCTACAACCGGGAGGAGTACGTGCGCTTCGACAGCGACGTGG
			ACGAGTACCGGCCGGTGACGGAGCTGGGCCGGCCGGACGCCGAGTACTGGAACCGCCAGCAGGACATCAT
			GGAGCGGAAGCGGGCCGAGCTGG
*Mibe*-DQB*U022	1	163	CAGCGGGTGCGGCTTGTGACCAGATACATCTACAACCGCGAGGAGTACGTGCGCTTCGACAGCGACGTGG
			GCGAGTACCGGGCCGTGACGCCGCTGGGCCGGCCGGACGCCGAGTACTGGAACCGCCAGCAGGACTTCCT
			GGAGCAGACGCGGGCCGAGCTGG

The number of individuals from which sequence was retrieved and length (bp) is given. Sequences retrieved from one individual only were not submitted to a public repository to ensure the storage of high quality sequences only
