# Supplementary material for: MHC class II variation in a rare and ecological specialist mouse lemur reveals lower allelic richness and contrasting selection patterns compared to a generalist and widespread sympatric congener
Source: Immunogenetics. 2015 Feb 18;67(4):229–45. doi: 10.1007/s00251-015-0827-4 (PMC4357647; doi:10.1007/s00251-015-0827-4)

**Electronic supplementary material**

**Article title:** MHC class II variation in a rare and ecological specialist mouse lemur reveals lower allelic richness and contrasting selection patterns compared to a generalist and widespread sympatric congener

**Journal name:** Immunogenetics

**Authors names:** Eva Pechouskova^1^, Melanie Dammhahn^2^, Markus Brameier^3^, Claudia Fichtel^1^, Peter M. Kappeler^1,4^* and Elise Huchard^5^*

**Affiliations:** ^1^*Behavioral Ecology and Sociobiology Unit, German Primate Center, Kellnerweg 4, Göttingen,Germany; ^2^Animal Ecology, University of Potsdam, Maulbeerallee 1, 14469 Potsdam, Germany; ^3^Primate Genetics Laboratory, German Primate Center, Kellnerweg 4, Göttingen, Germany ; ^4^Department of Sociobiology/Anthropology, Johann-Friedrich-Blumenbach Institut für Zoologie & Anthropologie Universität Göttingen, Kellnerweg 6, 37077 Göttingen, Germany; ^5^CEFE-CNRS, 1919 Route de Mende, 34295 Montpellier Cedex 5, France.*

**Corresponding author:** Eva Pechouskova – emailto: [epechouskova@dpz.eu](mailto:epechouskova@dpz.eu), Tel: +49551 3851 468

**Fig. ESM 1** Sample size distribution of newly captured individuals within three study areas (N5, Savannah and CS7) between the years 2005-2013 (2010 for Savannah). Only individuals included in the downstream analyses are shown. Trapping sessions for N5 were conducted 6-10 times a year and less regularly in Savannah and within the area of the known species distribution in CS7.


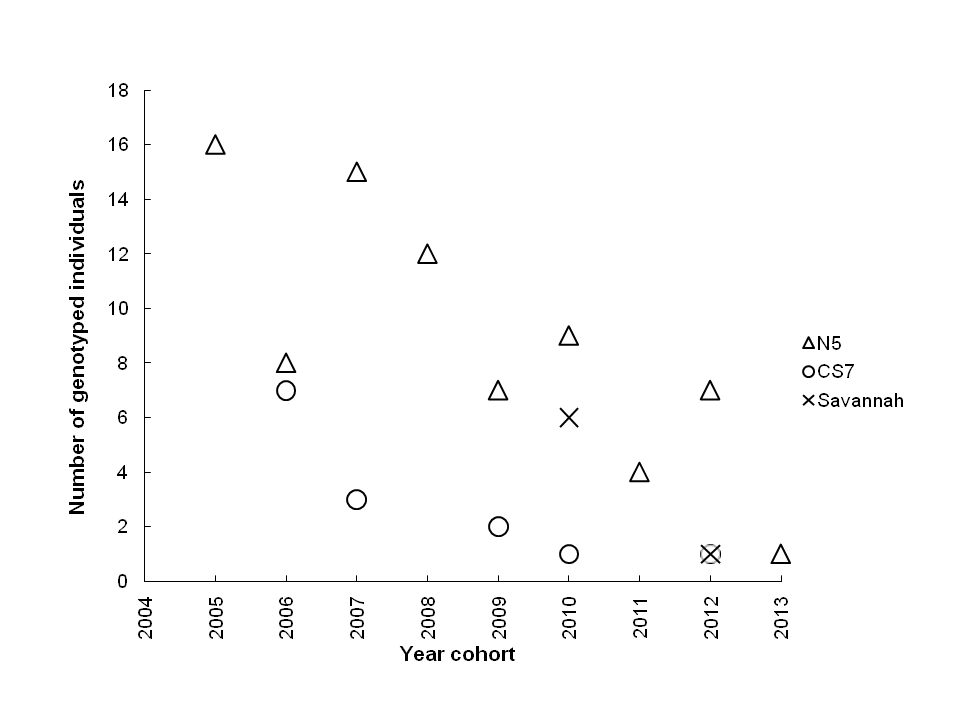

Supplement: Supplementary file 1 — (DOCX 79 kb) [file 251_2015_827_MOESM1_ESM.docx]
