# Supplementary material for: MHC class II variation in a rare and ecological specialist mouse lemur reveals lower allelic richness and contrasting selection patterns compared to a generalist and widespread sympatric congener
Source: Immunogenetics. 2015 Feb 18;67(4):229–45. doi: 10.1007/s00251-015-0827-4 (PMC4357647; doi:10.1007/s00251-015-0827-4)
Supplement: Supplementary file 2 — (DOCX 14 kb) [file 251_2015_827_MOESM2_ESM.docx]

**Table ESM 2** PCR reaction mix and conditions.

|  | ***Roche High Fidelity PCR system*** |
| --- | --- |
| ***PCR reaction mix*** | 2.5µl Buffer; 1.8mM MgCl2; 0.5 dNTPs; |
|  | 0.25 High Fidelity Taq Polymerase; |
|  | 2µl of genomic DNA template. |

|  |  | Temperature | Time |
| --- | --- | --- | --- |
| ***PCR conditions*** | 1x | 95°C | 2 min |
|  | 35x | 60^1^-62^2^°C | 30 s |
|  |  | 72°C | 45 s |
|  | 1x | 72°C | 7 min |

^1^DRB; ^2^DQB
