# Supplementary material for: MHC class II variation in a rare and ecological specialist mouse lemur reveals lower allelic richness and contrasting selection patterns compared to a generalist and widespread sympatric congener
Source: Immunogenetics. 2015 Feb 18;67(4):229–45. doi: 10.1007/s00251-015-0827-4 (PMC4357647; doi:10.1007/s00251-015-0827-4)
Supplement: Supplementary file 3 — (DOCX 12 kb) [file 251_2015_827_MOESM3_ESM.docx]

**Table ESM 3** Summary statistics of sequencing outcome for all reads passing the initial quality control procedure that required: i) a minimum of five reads for each sequence within individual amplicon, ii) a minimum two different sequences per amplicon, iii) minimum sequence frequency of 5% among all sequences of an amplicon (Huchard et al. 2012).

|  | ***DRB*** | ***DQB*** |
| --- | --- | --- |
| ***Nr_reads per amplicon*** |  |  |
| Mean±SD | 302.50±34.64 | 184.20±16.68 |
| Median | 169.5 | 101.5 |
| Min-max | 5 - 2255 | 5 - 832 |
|  |  |  |
| ***Nr_sequences per amplicon*** |  |  |
| Mean ±SD | 44.51±0.98 | 2.72±0.18 |
| Median | 2 | 2 |
| Min-max | 1 - 130 | 1 - 14 |
|  |  |  |
| ***Total amplicons*** | 148 | 130 |
| ***Total individuals*** | 96 | 98 |
| ***Total reads*** | 44 770 | 23 948 |
