# Supplementary material for: MHC class II variation in a rare and ecological specialist mouse lemur reveals lower allelic richness and contrasting selection patterns compared to a generalist and widespread sympatric congener
Source: Immunogenetics. 2015 Feb 18;67(4):229–45. doi: 10.1007/s00251-015-0827-4 (PMC4357647; doi:10.1007/s00251-015-0827-4)
Supplement: Supplementary file 4 — (DOCX 172 kb) [file 251_2015_827_MOESM4_ESM.docx]

**Fig. ESM 4** Distribution of relative per amplicon frequency (RPAF) for DRB (a) and DQB (b) and mean per-amplicon frequency (MPAF) for DRB (c) and DQB (d) of the first to sixth most common variants across amplicons and TMCA (two most common variants) and RA (remaining variants)

**
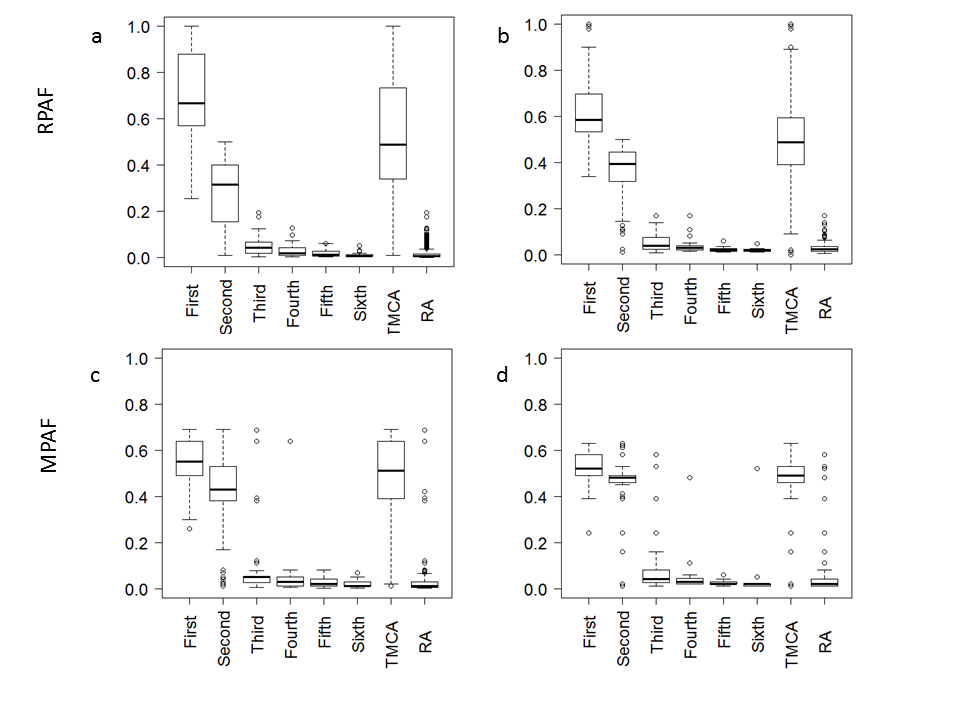
**
