# Supplementary material for: MHC class II variation in a rare and ecological specialist mouse lemur reveals lower allelic richness and contrasting selection patterns compared to a generalist and widespread sympatric congener
Source: Immunogenetics. 2015 Feb 18;67(4):229–45. doi: 10.1007/s00251-015-0827-4 (PMC4357647; doi:10.1007/s00251-015-0827-4)

**Fig. ESM 5** Mean per amplicon frequency (MPAF) in relation to allelic frequency (number of individuals carrying a given allele) for DRB alleles before (a) and after (b) allele sorting and for DQB alleles before (c) and after (d) allele sorting


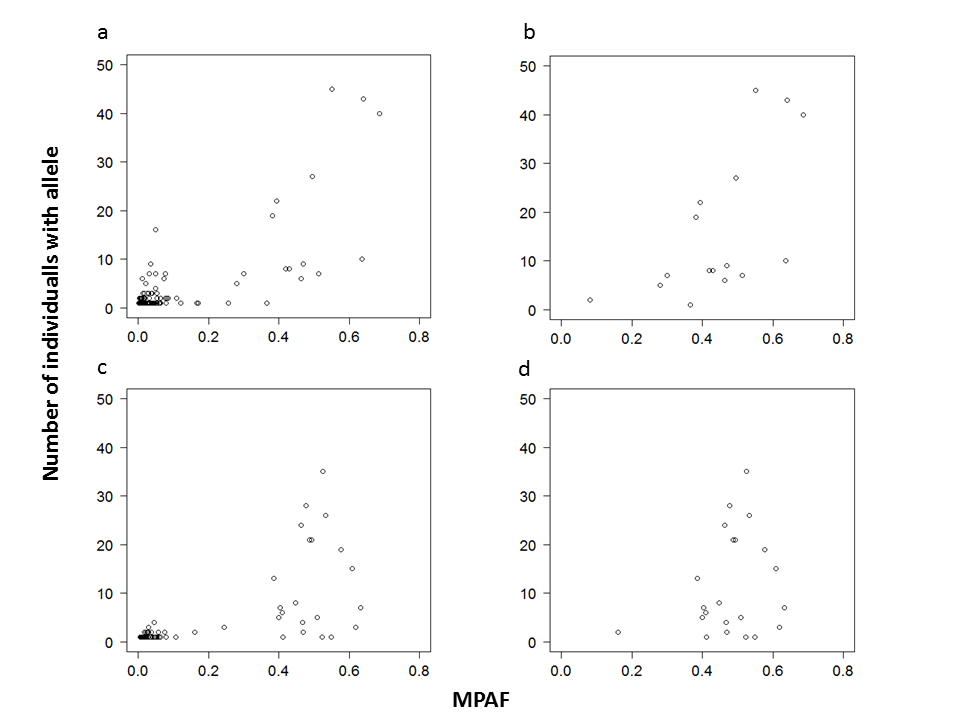

Supplement: Supplementary file 5 — (DOCX 124 kb) [file 251_2015_827_MOESM5_ESM.docx]
