# Supplementary material for: MHC class II variation in a rare and ecological specialist mouse lemur reveals lower allelic richness and contrasting selection patterns compared to a generalist and widespread sympatric congener
Source: Immunogenetics. 2015 Feb 18;67(4):229–45. doi: 10.1007/s00251-015-0827-4 (PMC4357647; doi:10.1007/s00251-015-0827-4)
Supplement: Supplementary file 6 — (DOCX 22 kb) [file 251_2015_827_MOESM6_ESM.docx]

**Table ESM 6** The overview of the allelic frequency distribution within each sub-population N5, Savannah and CS7 (DQB: a, DRB: b) and across year cohorts 2005-2013 (DQB: c, DRB: d) within the largest sub-population (N5). Sequences *Mibe*-DQB*U018, *U019, *U020, *U021, *U022 and *Mibe*-DRB U017 were found in one individual only.

**a.**

| **Allele_DQB** | **N5** | **Savannah** | **CS7** |
| --- | --- | --- | --- |
| *Mibe*-DQB*001 | 0.14 | 0.10 | 0.11 |
| *Mibe*-DQB*002 | 0.13 | 0.20 | 0.04 |
| *Mibe*-DQB*003 | 0.12 | - | 0.07 |
| *Mibe*-DQB*004 | 0.08 | 0.20 | 0.18 |
| *Mibe*-DQB*005 | 0.09 | 0.10 | 0.11 |
| *Mibe*-DQB*006 | 0.10 | - | 0.04 |
| *Mibe*-DQB*007 | 0.06 | - | 0.07 |
| *Mibe*-DQB*008 | 0.06 | - | 0.11 |
| *Mibe*-DQB*009 | 0.04 | 0.10 | - |
| *Mibe*-DQB*010 | 0.02 | 0.10 | 0.07 |
| *Mibe*-DQB*011 | 0.03 | - | 0.04 |
| *Mibe*-DQB*012 | 0.03 | - | - |
| *Mibe*-DQB*013 | 0.03 | - | 0.04 |
| *Mibe*-DQB*014 | 0.02 | 0.10 | - |
| *Mibe*-DQB*015 | 0.01 | - | 0.07 |
| *Mibe*-DQB*016 | 0.03 | - | - |
| *Mibe*-DQB*017 | 0.01 | 0.10 | - |
| *Mibe*-DQB*U018 | - | - | 0.04 |
| *Mibe*-DQB*U019 | - | - | 0.04 |
| *Mibe*-DQB*U020 | 0.01 | - | - |
| *Mibe*-DQB*U021 | 0.01 | - | - |
| *Mibe*-DQB*U022 | 0.01 | - | - |

**b.**

| **Allele_DRB** | **N5** | **Savannah** | **CS7** |
| --- | --- | --- | --- |
| *Mibe*-DRB*001 | 0.19 | - | 0.19 |
| *Mibe*-DRB*002 | 0.16 | 0.10 | 0.12 |
| *Mibe*-DRB*003 | 0.14 | 0.10 | 0.08 |
| *Mibe*-DRB*004 | 0.12 | 0.20 | 0.04 |
| *Mibe*-DRB*005 | 0.07 | 0.10 | 0.15 |
| *Mibe*-DRB*006 | 0.06 | - | 0.12 |
| *Mibe*-DRB*007 | 0.03 | 0.10 | 0.04 |
| *Mibe*-DRB*008 | 0.03 | 0.10 | 0.08 |
| *Mibe*-DRB*009 | 0.03 | 0.10 | 0.04 |
| *Mibe*-DRB*010 | 0.03 | - | 0.04 |
| *Mibe*-DRB*011 | 0.03 | - | 0.04 |
| *Mibe*-DRB*012 | 0.03 | - | - |
| *Mibe*-DRB*013 | 0.03 | 0.10 | - |
| *Mibe*-DRB*014 | 0.01 | - | 0.08 |
| *Mibe*-DRB*015 | 0.02 | 0.10 | - |
| *Mibe*-DRB*016 | 0.01 | - | - |
| *Mibe*-DRB*U017 | 0.01 | - | - |

**c.**

| **Allele_DQB** | **2005** | **2006** | **2007** | **2008** | **2009** | **2010** | **2011** | **2012** | **2013** |
| --- | --- | --- | --- | --- | --- | --- | --- | --- | --- |
| *Mibe*-DQB*001 | 0.06 | 0.13 | 0.20 | 0.21 | 0.07 | 0.22 | 0.25 | - | - |
| *Mibe*-DQB*002 | 0.09 | 0.13 | 0.10 | 0.21 | 0.07 | 0.11 | 0.13 | 0.21 | - |
| *Mibe*-DQB*003 | 0.13 | 0.13 | 0.20 | 0.04 | 0.07 | 0.11 | 0.13 | 0.14 | - |
| *Mibe*-DQB*004 | 0.09 | 0.13 | 0.10 | 0.04 | 0.14 | - | - | - | 0.50 |
| *Mibe*-DQB*005 | 0.13 | - | 0.03 | 0.13 | 0.21 | 0.06 | - | 0.14 | - |
| *Mibe*-DQB*006 | 0.09 | 0.13 | - | 0.13 | - | 0.11 | 0.13 | 0.29 | 0.50 |
| *Mibe*-DQB*007 | 0.09 | 0.06 | 0.03 | 0.04 | 0.14 | 0.06 | - | - | - |
| *Mibe*-DQB*008 | 0.03 | 0.06 | 0.07 | 0.04 | 0.07 | 0.06 | - | 0.21 | - |
| *Mibe*-DQB*009 | 0.06 | - | - | 0.04 | - | - | 0.38 | - | - |
| *Mibe*-DQB*010 | 0.03 | 0.06 | 0.03 | - | - | - | - | - | - |
| *Mibe*-DQB*011 | 0.09 | - | 0.03 | - | - | 0.06 | - | - | - |
| *Mibe*-DQB*012 | 0.03 | 0.06 | 0.03 | 0.04 | 0.07 | - | - | - | - |
| *Mibe*-DQB*013 | - | - | 0.07 | - | - | 0.17 | - | - | - |
| *Mibe*-DQB*014 | 0.03 | - | - | 0.04 | - | 0.06 | - | - | - |
| *Mibe*-DQB*015 | - | - | - | - | 0.07 | - | - | - | - |
| *Mibe*-DQB*016 | - | 0.06 | 0.07 | - | 0.07 | - | - | - | - |
| *Mibe*-DQB*017 | 0.03 | - | - | - | - | - | - | - | - |
| *Mibe*-DQB*U018 | - | - | - | - | - | - | - | - | - |
| *Mibe*-DQB*U019 | - | - | - | - | - | - | - | - | - |
| *Mibe*-DQB*U020 | - | 0.06 | - | - | - | - | - | - | - |
| *Mibe*-DQB*U021 | - | - | 0.03 | - | - | - | - | - | - |
| *Mibe*-DQB*U022 | - | - | - | 0.04 | - | - | - | - | - |

**d.**

| **Allele_DRB** | **2005** | **2006** | | **2007** | | **2008** | | **2009** | | **2010** | | **2011** | | **2012** | | **2013** | |  |
| --- | --- | --- | --- | --- | --- | --- | --- | --- | --- | --- | --- | --- | --- | --- | --- | --- | --- | --- |
| *Mibe*-DRB*001 | 0.22 | | 0.13 | | 0.03 | | 0.25 | | 0.3 | | 0.17 | | 0.13 | | 0.43 | | 0.25 | |
| *Mibe*-DRB*002 | 0.19 | | 0.13 | | 0.2 | | 0.08 | | 0.1 | | 0.11 | | 0.5 | | 0.14 | | - | |
| *Mibe*-DRB*003 | 0.06 | | 0.13 | | 0.2 | | 0.21 | | - | | 0.22 | | 0.25 | | - | | 0.25 | |
| *Mibe*-DRB*004 | 0.09 | | 0.13 | | 0.1 | | 0.21 | | - | | 0.11 | | 0.13 | | 0.21 | | - | |
| *Mibe*-DRB*005 | 0.09 | | 0.13 | | 0.1 | | 0.08 | | 0.1 | | - | | - | | - | | - | |
| *Mibe*-DRB*006 | 0.03 | | 0.06 | | 0.07 | | 0.04 | | - | | 0.06 | | - | | 0.21 | | - | |
| *Mibe*-DRB*007 | 0.09 | | - | | - | | 0.04 | | 0.1 | | - | | - | | - | | - | |
| *Mibe*-DRB*008 | 0.03 | | 0.06 | | 0.03 | | - | | - | | - | | - | | - | | 0.25 | |
| *Mibe*-DRB*009 | - | | 0.06 | | 0.03 | | - | | 0.1 | | 0.06 | | - | | - | | - | |
| *Mibe*-DRB*010 | 0.09 | | - | | 0.03 | | - | | - | | 0.06 | | - | | - | | - | |
| *Mibe*-DRB*011 | - | | - | | 0.07 | | - | | - | | 0.17 | | - | | - | | - | |
| *Mibe*-DRB*012 | 0.03 | | 0.06 | | 0.03 | | 0.04 | | 0.1 | | - | | - | | - | | - | |
| *Mibe*-DRB*013 | 0.03 | | 0.06 | | 0.07 | | - | | - | | - | | - | | - | | - | |
| *Mibe*-DRB*014 | - | | 0.06 | | - | | - | | 0.1 | | - | | - | | - | | - | |
| *Mibe*-DRB*015 | 0.03 | | - | | - | | 0.04 | | - | | 0.06 | | - | | - | | - | |
| *Mibe*-DRB*016 | - | | - | | - | | - | | 0.1 | | - | | - | | - | | 0.25 | |
| *Mibe*-DRB*U017 | - | | - | | 0.03 | | - | | - | | - | | - | | - | | - | |
